# A qualitative meta-synthesis of patient dignity from the perspective of caregivers

**DOI:** 10.1186/s12877-023-04071-1

**Published:** 2023-06-06

**Authors:** Minyu Liang, Xiyan Xie, Yichao Pan, Andy S. K. Cheng, Zengjie Ye

**Affiliations:** 1 School of Nursing, Guangzhou University of Chinese Medicine, Guangzhou, Guangdong Province China; 2 Nursing Department, Home for the Aged Guangzhou, Guangzhou, Guangdong Province China; 3 Department of Cardiovascular Medicine, Guangzhou First People’s Hospital, Guangzhou, Guangdong Province China; 4 Department of Rehabilitation Sciences, The Hong Kong Polytechnic University, Hong Kong, China

**Keywords:** Dignity, Caregivers’ perspective, Literature review, Qualitative evidence

## Abstract

**Background:**

The concept of dignity remains disputed, with most studies defining dignity based on its external dimension. Although its inherent dimension is a rooted attribute of dignity, it has received scarce attention. Caregivers have close relationships with their care recipients and thus may perceive their patient’s inherent as well as external dimensions of dignity. Therefore, in this study, we aimed to identify, analyze, and synthesize evidence on human dignity presented in qualitative studies from the perspective of caregivers to gain a deeper comprehension of the preservation of patients’ dignity by their caregivers.

**Methods:**

A qualitative meta-synthesis was performed by searching for relevant qualitative literature via systematic electronic databases, including MEDLINE, PsycINFO, ProQuest, CINAHL, Embase, Health Source, and Web of Science, from inception to March 15, 2022.

**Results:**

Nine studies were eligible for inclusion and included in the meta-synthesis. Three overarching categories were identified: integrated person, “rootedness” and “growth” atmosphere, and balanced state.

**Conclusions:**

Dignity is rooted in its inherent dimension, whereas its external dimension may promote individual dignity. Furthermore, caregiver-patient relationships may be a key factor linking the inherent dimension of dignity with its external dimension. Thus, further studies should focus on the mechanism of relationships in preserving dignity.

## Background

The concept of dignity is anchored in the different rules of conduct and guidelines relating to patient care [1]. Additionally, many international organizations and countries have applied the concept of human dignity as a central theme in political agendas, health policy decisions, and public health ethics. The Declaration of Human Rights and the International Council of Nurses (ICN) Code of Ethics highlight the intrinsic properties of dignity as a fundamental human right [2, 3]. Furthermore, the German constitution contains an article on the inviolability of human dignity, illustrating the importance of dignity in Germany’s health policies on disease prevention and health promotion [4]. Similarly, Norway, Finland, Sweden, and Denmark have implemented legislations on dignity by emphasizing the right to a dignified life for older citizens, including a safe and meaningful experience of life and well-being [5–8].

Although the term dignity is used in various codes of conduct, guidelines, and criteria for patient care, the concept of dignity continues to be debated because individual perceptions of the attributes of dignity differ based on the perception factors [9]. A literature analysis by Chochinov et al. devised a dignity-conserving care model. for the terminally ill based on three main categories: illness-related concerns, a dignity-conserving repertoire, and a social dignity inventory [10]. These categories covered varied factors, including physical, psychosocial, spiritual, and existential characteristics. Another study by Nordenfelt et al. constructed four themes of dignity in older adults: the dignity of merit, dignity as moral stature, the dignity of identity, and human dignity [11]. Human dignity is considered to consist of two dimensions: the inherent dimension and the external dimension. The inherent dimension of dignity refers to a sense of self-worth and is perceived as an intrinsic characteristic of humans [12]. This dimension is grounded in people’s beliefs, rooted in their values and perceptions, and expressed as feelings of self-esteem, personal valuation, and being good [12]. The external dimension of dignity represents the social and cultural life that can be promoted or violated through validation by others and is a scalable dimension [12]. Thus, most studies define dignity using its external dimension and underline the importance of the social aspect obtained from others to maintain dignity [9, 13–15]. Nevertheless, the inherent dimension of dignity is a rooted attribute that can be fostered by the external dimension. Thus, dignity can be emphasized through its inherent dimension and its relation with the external dimension. Caregivers are actively committed to the physical and psychological care of their patients. Caregivers involved in their patient’s life may attain an understanding of their patient’s dignity from the inherent dimension, and their care relationship will help elevate patient dignity from the social aspect (i.e., the external dimension). Therefore, a caregiver’s perspective of dignity may significantly affect the preservation of their patient’s inherent and external dimensions of dignity. However, most previous studies have explored dignity from the perspectives of patients and medical professionals [16–19]. Only a few studies have explored dignity from the caregivers’ perspective, wherein most researchers have explored its meaning from the opposite viewpoint, i.e., indignity [20–22]. In these researches, the violation of dignity was commonly examined, ranging from stereotyping to the inattentive attitudes of caregivers when providing care [21, 22]. Suffering, distrust, a feeling of inferiority, and humiliation among patients may result from the caregivers’ lack of respect toward their patients [22]. Moreover, certain caregivers regularly provide care without reflecting on their actions, which may affect the care quality provided. However, limited studies have investigated the individual value that underlies this behavior. Therefore, further research would benefit by studying the values that affect caregivers’ decision-making while providing care. Furthermore, dignity as a value indicator that influences individual behavior should be focused upon. The perception of dignity by caregivers may determine their care value and regulate their caring behavior, which could contribute to the dignity-conserving care model and positively affect patient health outcomes. Based on the discussed literature, our study aimed to synthesize the meaning of dignity from the caregivers’ perspective, which may help decipher the relation between the inherent and external dimensions of dignity.

### Theoretical framework of the study

We employed Florence Nightingale’s environmental theory as a theoretical framework for the research data. According to this theory, a patient’s condition and nature are interrelated with their physical, psychological, and social environment [23]. In 1860, Nightingale first promulgated dignity as a factor within the environment and noted its active role in restoring one’s vitality; however, its meaning was not explained in detail [24]. Thus, we further explored the concept of dignity within the framework of Nightingale’s environmental theory that a person’s nature interacts with the physical, psychological, and social environment.

## Method

### Study design

A qualitative meta-synthesis was performed to explore caregivers’ perspectives of dignity in care. This comparative and comprehensive qualitative research method yields a rich and extensive understanding the integrity of a given phenomenon. Compared with individual studies, a meta-synthesis presents results with advanced coherence, cogency, and utility that help inform pragmatic decisions. Additionally, we used the ENTREQ statement to improve transparency in reporting the synthesis of our qualitative research [25]. Finally, the Critical Appraisal Skills Program (CASP) [26] and thematic synthesis [27] were applied to appraise the quality of the qualitative evidence.

### Search strategy

Qualitative literature relevant to the study was searched using systematic electronic databases, including MEDLINE, PsycINFO, ProQuest, CINAHL, Embase, Health Source, and Web of Science, from inception to March 15, 2022. The primary research tactics were based on PICo (Population, Interest, and Context). The retrieved items were as follows: caregivers, family members, and relatives (population); perceptions of dignity, perspectives of dignity, and meanings of dignity (interest); and care of patients, care of family members, and care quality (context). Titles and abstracts were initially screened for relevance by two authors, who then reviewed the pertinent full text of the selected papers. Additionally, any differing opinions were discussed and sent to a third author if an agreement could not be reached.

### Selection criteria

Papers were selected if they (a) employed qualitative methods in the data collection and analysis, (b) presented in English and published in a peer-reviewed journal, and (c) illustrated the dignity of individuals from the caregiver’s perspective. Furthermore, dignity should have been explicitly emphasized as a main theme in the primary research or a part of the study outcomes. Papers were excluded if (a) their findings did not contain content to realize the synthesis (i.e., no theme reported), (b) their results described caregivers’ experience without their views on maintaining dignity, or (c) caregivers were medical workers.

### Quality assessment

The CASP qualitative research checklist was applied by two researchers to assess the quality of the included papers. If any differences arose, they were discussed and sent to a third reviewer (if unresolved after discussion) to obtain a consensus. CASP was used to measure the bias risk and quality of the included studies in the following areas: the research aim, methodology, research design, data collection, recruitment, data analysis, ethical issues, value, relationship, and research findings. Scores were assigned for each paper, ranging from 0–10. A score of 9–10 indicated a high-quality paper, 7.5–9 represented a moderate-quality paper, and < 7.5 indicated a low-quality paper. Studies that scored < 6 were excluded [28].

### Data synthesis

The synthesis was performed based on the principle of the interpretative synthesis approach [29], with an aim to develop the concept further and elaborate the integrated concepts or relative theory in the original research [30]. The collected data were analyzed and synthesized by employing a qualitative content analysis technique that constituted the following six steps [31]. (1) All authors read the included studies to understand the whole research. The text about dignity was then extracted as meaning units by two researchers (LMY and XXY). (2) The meaning units were further condensed by the two researchers (LMY and XXY) to assign the extracted meaning units using their own language. (3) Next, two researchers (PYC and ASKC) labeled the condensed meaning units using codes. (4) These codes were compared by all researchers to identify the differences and similarities. (5) Further, the codes were sorted into subcategories that were eventually organized into categories. (6) Finally, in the comprehensive understanding phase, a process of reflection on the analysis procedure and results analysis was performed by all researchers to develop an integrated latent theme [31].

## Result

### Study characteristics

**Fig. 1:**
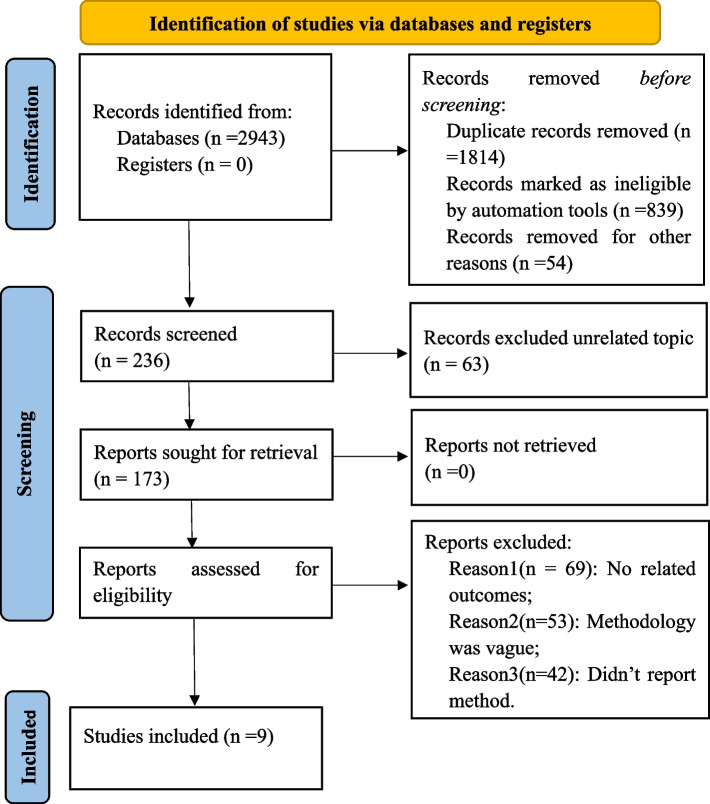
Literature review flowchart

Among the 2943 records screened, nine studies met the inclusion criteria and were used for further quality evaluation [20, 32–39]. (Fig. 1). Table 1 describes the features of the included studies. All studies included caregivers as the participants. Most studies (6/9) explored dignity in family caregivers or relatives, while the remaining studies enrolled non-professional caregivers and did not clarify their relationship with the care receivers. Most research was conducted in the Occident (*n* = 7), while the remaining studies originated from Asia (*n* = 2).

**Table 1:** Characteristics of included studies

**Study**	**Aim**	**Demographic**		**Methods**				**Results**
		**N**	**sample**	**Methodology**	**Data collection**	**Data analysis**		
Kalis et al. 2005 [32]	Perspective of a good life	10	Caregivers	Grounded theory	Observation, unstructured interviews, focus-group, individual interview	Content analysis		Category1:peace and quiet Category2:going along with subjective experience Category3:no enforcement
Anderson et al. 2021[33]	Concept of dignity	9	Family caregivers	Qualitative descriptive study	A purposive sample of blogs	Content analysis		Category1:perceived value from others Category2:self in relation to others Category3:behavioral respect, and self-value
Tehranineshat et al. 2020[34]	Experiences and perceptions of patient dignity	8	Family caregivers	Descriptive phenomenological approach	Semi-structured, in- depth interviews	Content analysis		Category1:a peaceful environment Category2:respect Category3:comprehensive support
Gharibian et al. 2015[35]	The perspectives of quality of life	19	Family caregivers and care staff	Grounded theory	Unstructured interviews	Glaser & Strauss classic grounded theory		Theme1:maintaining family connectedness Theme2:engaging in worthwhile activities Theme3:maintaining and developing significant relationships Theme4:holding and practicing spiritual beliefs
Melin-Johansson et al. 2007[36]	Perceptions about terminally ill family members	4	Caregivers	Qualitative descriptive study	Semi-structured interviews	Content analysis		Category1:living a normal life Category2:being relieved from burdens Category3:having a sense of belonging Category4:being a symbol of incurable illness Category5:haiving a sense of dignity
Nåden et al. 2013 [20]	Knowledge about maintaining dignity in nursing homes	28	Family caregivers	Phenomenological hermeneutic method	Semi-structured interviews, individual interview	Hermeneutics analysis		Theme: A feeling of being abandoned Subtheme1:deprived of the feeling of belonging Subtheme2:deprived of dignity due to acts of omission, deprived of confirmation Subtheme3:deprived of dignity due to physical humiliation, Subtheme4:deprived of dignity due to psychological humiliation Subtheme5:deprived of parts of life
Caspari et al. 2014[37]	Experience of nursing home residents’ dignity	28	Relatives	Phenomenological hermeneutic method	Semi-structured interviews, individual interview	Hermeneutics analysis		Theme1:to have a comfortable, homely and practical room Theme2:to have close contact with family, friends and with the staff Theme3:to have aesthetic needs and concerns attended to Theme4:to have ethical needs and intrinsic values attended to Theme5:to have cultural and spiritual needs and concerns attended to
Rehnsfeldt et al. 2014)[38]	The meaning of dignity in nursing home	28	Relatives	Hermeneutic Epistemology	In-depth interviews	Hermeneutics analysis		Theme1:dignity as at-home-ness, dignity Theme2:the little extra. Theme3:nondignifying ethical context
Lou et al. 2021 [39]	The meaning of dignity	31	Caregivers	Descriptive phenomenological approach	Semi-structured interviews, group interviews	Content analysis		Theme1:illness related concerns Theme2:dignity conserving repertoire Theme3:social dignity inventory

### Methodological quality

The evaluation of each study’s quality is presented in Table 2. The CASP checklist scores of the papers ranged from 8 to 10, indicating that the included studies were of moderate to high quality. Furthermore, the CASP results showed that the researcher and patient relationship was not elaborated in most included studies (88.9%), reducing the study quality. Lastly, the models of individual dignity that were used concentrated on affecting factors and attributes from the perspective of caregivers.

**Table 2:** Results of CASP quality appraisal

	**Studies**	**Q1**	**Q2**	**Q3**	**Q4**	**Q5**	**Q6**	**Q7**	**Q8**	**Q9**	**Q10**	**Total score**	**Quality rating**
1	Kalis et al. 2005 [32]	Y	Y	Y	U	Y	N	Y	Y	Y	Y	8.5	MED
2	Anderson et al. 2021[33]	Y	Y	Y	U	Y	N	Y	Y	Y	Y	8.5	MED
3	Tehranineshat et al. 2020[34]	Y	Y	Y	Y	Y	Y	Y	Y	Y	Y	10	HIGH
4	Gharibian et al. 2015[35]	Y	Y	Y	Y	Y	N	Y	Y	Y	Y	9	HIGH
5	Melin-Johansson et al. 2007[36]	Y	Y	Y	U	Y	N	Y	Y	Y	Y	8.5	MED
6	Nåden et al. 2013 [20]	Y	Y	Y	Y	Y	N	Y	Y	Y	Y	9	HIGH
7	Caspari et al. 2014[37]	Y	Y	Y	U	Y	N	Y	Y	Y	Y	8.5	MED
8	Rehnsfeldt et al. 2014)[38]	Y	Y	Y	U	Y	N	Y	U	Y	Y	8	MED
9	Lou et al. 2021 [39]	Y	Y	Y	Y	Y	U	Y	U	Y	Y	9	HIGH

Notes:

Y = a rating of “yes”; U = a rating of “unclear”, N = a rating of “no”

Scoring system: Y = 1 point, U = 0.5, No = 0 point

High quality 9–10; moderate quality 7.5–9; low quality < 7.5; exclude < 6

Q1: Was there a clear statement of the aims of the research?

Q2: Is a qualitative methodology appropriate?

Q3: Was the research design appropriate to address the aims of the research?

Q4: Was the recruitment strategy appropriate to the aims of the research?

participants

Q5: Was the data collected in a way that addressed the research issue?

Q6: Has the relationship between researcher and participants been adequately considered?

Q7: Have ethical issues been taken into consideration?

Q8: Was the data analysis sufficiently rigorous?

Q9: Is there a clear statement of findings?

Q10: How valuable is the research?

### Meta-synthesis

**Fig. 2:**
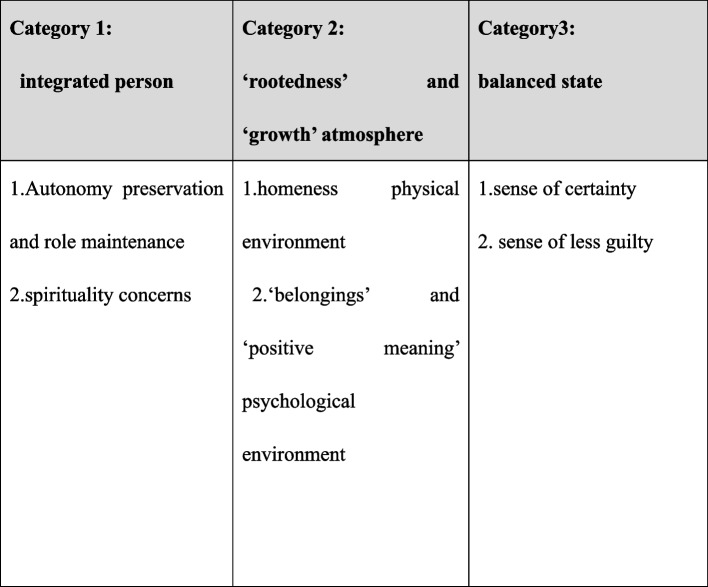
Model structure of patient dignity from the caregivers’ perspective

Our meta-synthesis identified three categories: integrated person, ‘rootedness’ and ‘growth’ atmosphere, and balanced state. The model structure of patient dignity is elaborated in Fig. 2. Additionally, exemplar coding and extracts of the original studies for each descriptive category of meta-synthesis is illustrated in Table 3.

**Table 3:** Synthesized categories: an overview of categories and subcategories with examples of condensed meaning units

Category	Integrated person	“Rootedness” and “growth” atmosphere	Balanced state
Subcategories	Autonomy preservation and role maintenance Spirituality concerns	“Homeness” physical environment and “belonging” and “positive meaning” psychological environment	Sense of certainty ***Sense of*** less guilt
Condensed meaning units	Being significant by maintaining a normal life and participating in social life [36] Wanting to remain an individual with her/his demands [36] Desiring to be treated as a whole person even when terminally ill [36] Bolstering of dignity when being able to do something for themselves or others [39] Bolstering of dignity when being recognized as an individual separate from the illness [39] Toning down their needs [36] Independently caring for themselves to be considered as a person [33] Needing to preserve identity and integrity was important [37] Wanting to participate in life [36] No invitation or engagement leads to a dignity-depriving situation [20] Distress arises because of inability to perform everyday activities [39] Involving patients in advanced care planning strengthens their dignity [39] Being respected when being able to sustain their will to manage their life [32] Respecting the patient’s decision was the best way to express filial piety [39] Respecting the involvement of patients in decision-making enhanced patients’ values [34] Desiring to make decisions of daily life management even when terminally ill [37] Providing care without considering a person’s capabilities violates a patient’s dignity [38] Upholding and practicing spiritual beliefs for elevating strength [35] Seeking spiritual comfort and relying on the sovereignty of a higher power, e.g., a Buddhist patient [39] Regarding religious faith as spiritual needs [37] Having no opportunity to express religious belief [37] Respecting patients’ beliefs [34] Putting some money in a charity box to maintain dignity [34]	Preferring to have a room with a private bathroom [37] Preserving a clean and tidy environment [37] Decorating the room with familial equipment and creating a warm atmosphere promotes a feeling of dignity [38] Preferring to have a room with their furniture and belongings [37] Feeling more dignified when receiving care at home [39] Home gives feelings of warmth, coziness, and safety [32] Strong relationships with descendants were identified as a source of hope, meaning, and pride [39] Grandchildren and great-grandchildren bring immense love and joy into life [36] Feeling at home by being close to relatives and caregivers [38] Caregivers perform small acts to establish a close connection with patients [38] Eagerness to have close connections with family, friends, and staff [37] Focus on relationships and social networks [32] Close bonds with others create a feeling of value and strength [35] Contentment and expressing thankfulness strengthen the positive sense of self [39] Positive expression increases the sense of self-worth [33] Feeling that illness provides openness and intimacy in their relationship with family members [36]	Illness-related concerns affecting dignity [39] Insufficient information about disease and treatment affecting the sense of dignity [34] Medical uncertainty creating psychological distress [39] Distress caused by an inability to purchase certain medications [34] Experiencing high stress levels and psychological tension when diagnosed with a disease and lack of treatment and follow-up care information [34] Feeling confident after gaining knowledge about medical information [37] Needing treatment teams to reduce the uncertainty [34] High stress levels and psychological tension requiring counseling services [34] Providing necessary medical instructions shows respect for patient dignity [34] Needing biographical knowledge incorporated into the care plan [35] Excessive care stress harms dignity [39] Relieving the caregiver from the care burden causes less sense of uselessness [36] Desiring resources to relieve care burden [39]

### Category 1: Integrated person

Integrated individual is centered on the bidirectional meaning of life encompassing a natural life and spiritual life [40]. Natural life represents human subjectivity, initiative, and self-reliance, whereas spiritual life refers to transcendence [1, 41]. In reference to this study, natural life relates to autonomy and role maintenance, while spiritual life denotes transcendence within the concept of religion.

#### Subcategory 1: Autonomy preservation and role maintenance

The preservation of autonomy and role maintenance was embodied in the patient’s desire to live a normal life, participate in worthwhile activities, and make decisions. Patients, including those who were terminally ill, did not want to be perceived as a person with an illness, but they desired to be treated as a whole individual and wished to manage their daily lives normally [36, 37]. Activities provided the patients with the feeling of concern for others as well as that of being useful and having autonomy [36, 38]. Additionally, patients, including terminally ill individuals, wanted to preserve their autonomy and decision-making role concerning their treatment plans and procedures. Moreover, caregivers highlighted that respecting a patient’s decision was the best way family members could express filial piety, whereas ignoring the patient’s will violated their dignity [38, 39]. Interestingly, one study reported that patients tended to tone down their needs to preserve autonomy [36]. Another study revealed that some family caregivers would satisfy their patients’ needs without reserve due to their sense of responsibility towards their patients [38].

#### Subcategory 2: Spirituality concerns

Spirituality was associated with the assertion of life via the patients’ relationship with self, the environment, and God. It can be interpreted as the impetus for meaning and a sense of purpose in life and serves as a source of renewal and emotional support, positively affecting health and fostering life dignity [35]. Furthermore, caregivers noted that spiritual belief endowed patients with hope, purpose, and control, with prayer and worship motivating emotional expression [39]. However, one study found that family members did not explicitly discuss their patients’ spiritual needs or concerns and limited their spiritual needs within the context of religious belief [37]. Nevertheless, caregivers provided their patients with religious songs and hymns to satisfy their spiritual needs [37]. It must be noted that this opportunity may be unavailable to patients with religious beliefs who reside among non-religious patients [37].

### Category 2: ‘Rootedness’ and ‘growth’ atmosphere

‘Rootedness’ and ‘growth’ atmosphere were perceived as an environment of dignity. Rootedness emphasizes comfort, security, belonging, and emotional connection with families, relatives, or friends [42, 43]. A growth atmosphere is experienced by patients who transform stressful events into a situation with a positive meaning [44], and this atmosphere can manifest in a physical and psychological environment.

#### Subcategory 1: Homeness physical environment

Considering that rootedness signified comfort and security, it could be explained as the sense of ‘homeness’ concerning the notion of home. In this study, the homeness physical environment is regarded as a private, aesthetic, and particularly ‘homelike’ condition. Moreover, caregivers have revealed that patients preferred to live in a room with a private bathroom [37], and patients could experience stress and problems with daily activities when sharing a room with a stranger [37]. Additionally, caregivers emphasized that cleaning the floors and tables, caring for the flowers, and maintaining a clean and comfortable home were extremely important, granting patients a sense of dignity [32, 38]. Caregivers also mentioned that patients preferred to equip their room with their furniture to experience ‘homeness’ and ‘belonging’ [37]. Finally, older people felt more dignified when accepting care at home than at a care institution [39].

#### Subcategory 2: ‘Belonging’ and ‘positive meaning’ psychological environment

Rootedness also entailed the property of belonging, which could be explained by the psychological environment of ‘belonging’. A feeling of belonging reflected a closely connected relationship with family members, relatives, and other caregivers. Caregivers reported that strong relationships with family members and respect from children and grandchildren provided a strong sense of belonging and served as a source of pride, meaning, and hope for their patients [35, 37, 39]. Moreover, patients wished to strengthen their relationships with relatives to enrich their sense of belonging [32, 38]. Finally, caregivers can closely connect with their patients by performing small deeds for them, such as shaking their hands or kneeling with the patient daily, thereby creating an intimate bond and conveying that the caregivers are truly interested in their patients’ welfare [38].

As mentioned, growth was interpreted as a positive meaning derived from stressful events. In this study, growth represented a great appreciation for life. Numerous studies have reported that caregivers noted that patients expressing thankfulness and reciprocating with courteousness and appreciation had an increased positive sense of self [33, 36, 39]. The caregivers also highlighted that strengthening familial bonds could facilitate collaboration and intimate conversations with family members, promoting the expression of emotional closeness, forgiveness, and gratitude [39].

### Category 3: Balanced state

Balanced state referred to the harmony and well-being of one’s mind [45], resulting from a sense of equilibrium between stressors and coping resources [46]. In our study, adequate social resources were considered the major factor required to maintain a balanced state.

#### Subcategory 1: Sense of certainty

Sense of certainty was one aspect related to a balanced state. Our findings suggested that adequate information was the key factor attributed to the certainty of disease and the role transfer of patients and caregivers. In contrast, caregivers expressed that patients experience a sense of uncertainty when provided with inadequate information. Caregivers further emphasized that patients regarded the provision of unsatisfactory medical information as a threat to their sense of dignity and related this to feelings of uncertainty when they were unaware of their medical and rehabilitation information. This uncertainty rendered the patients passive in coping with their disease and undermined their dignity [34, 36, 39]. Furthermore, patients and their family caregivers experienced high stress levels when the patients were diagnosed with diseases and developed a confused understanding of treatment processes and follow-up visits. Nevertheless, both of caregivers and patients requested professional help to identify their sources of stress and to assist them with adjusting to their current role [34, 37].

#### Subcategory 2: Sense of less guilt

The sense of less guilt was the second facet of a balanced state, wherein alleviating care pressures allowed the patients to feel less guilt. In contrast, our study found that family caregivers considered that being available round the clock to provide their patients with unconditional support was part of meeting their patients’ needs and being prepared for the worst. However, the heavy care burden perceived by the patients might stimulate their feelings of guilt [35]. Additionally, the caregivers might not discuss their care distress to alleviate their patients’ psychological burden [36]. Finally, the caregivers stressed the importance of social support organizations for supporting and relieving their care burden [36].

## Discussion

Our study is the first to employ the framework of Nightingale’s environmental theory to interpret the meaning of dignity, which could help explore the complex concept of dignity according to its multidimensions. Our findings revealed that the multidimensions of dignity could be elaborated into integrated person, ‘rootedness’ and ‘growth’ atmosphere, and balanced state.

Considering the patient as an integrated person is important in maintaining patient dignity. The concept of an integrated person includes a natural life and spiritual life, wherein natural life emphasizes preserving individual autonomy and role maintenance. Previous studies have highlighted that the satisfaction of autonomy and role maintenance generates an intrinsic value and translates into an improved sense of dignity, whereas the restriction of this ability may trigger a strong motivation in patients to exercise their autonomy and role, even by adopting a negative response [47–49]. These findings were consistent with those in our study. Although patients might be in a healthy condition, they prefer to be treated as a whole individual rather than a person with an illness, participating in activities and decision-making. Furthermore, our results demonstrated that patients would respond negatively when they could not maintain their autonomy and role. Additionally, some patients were prone to downplaying their needs and illnesses to avoid losing their freedom, becoming trapped, and feeling useless if they were to extend their needs and demands [36]. In contrast, some family caregivers provided help that exceeded their patients’ needs. These results indicate a great contradiction between care receiving and care providing, and a balance is required between them. Thus, caregivers should not only focus on providing care to meet their patients’ physical needs but should also consider their psychological requirements, particularly dignity. Recognizing the meaning of an integrated person is vital to establish a balance between providing care and preserving patients’ role and autonomy, thereby contributing to an effective dignity care model.

Spirituality is recognized as an inner resource for encouraging health maintenance and promotion, raising hope and resilience during illness distress, and improving patient outcomes [50, 51]. Cleland et al. proposed that spiritual comfort was the conceptual definition of dignity [52], with spiritual as well as psychosocial and physical elements included in the dignity-conserving model [53]. Patients’ spirituality is crucial to maintain dignity and ameliorating patient outcomes. In our study, family members acknowledged the importance of spiritual care in maintaining patient dignity; however, they tended to restrict the issue of spiritual care to religious beliefs. Our observation was consistent with a study reporting that the significance of respect for Iranian patients was rooted in their religious beliefs [54]. This was also in line with the research by Bayan et al. on 978 nurses that showed that the nurses regarded spiritual care as showing concern and respecting their patients’ religious beliefs [55]. Although religious coping, which is concerned with a constructive reliance on faith, has been shown to improve health adjustment and psychological adaptation to stressors and predict spiritual support, “spirituality” and “religion” have their distinctions. Spirituality is a multidimensional concept that involves seeking meaning in life and transcendence [56–58] and encompasses features more than those in the domain of religion. Spirituality is a component that assists an individual in realizing their true potential, promoting self-confidence, love, and forgiveness, and empowering them to transcend distress, ultimately leading to greater insight and inner peace regarded as a transcendent state [57]. For religious patients, the search for meaning in life and inner peace of mind could be supported by chaplains and clergy [59], whereas the spirituality of non-religious patients might be ignored. Therefore, other strategies are required to aid non-religious patients in achieving a transcendent state. For example, individuals can attain transcendence through art, music, nature, solidarity, meditation, mindfulness-based stress reduction techniques, physical activities (e.g., Tai chi or rhythmic movement), and integrative medicine (massage, aromatherapy, acupuncture, Rei Ki, and dietary supplements) [57, 60]. Caregivers could facilitate such non-religious measures for their patients to help them increase their spirituality. However, most caregivers in our study were not aware of the means to assist their patients in reaching a transcendent state. Therefore, we suggest that caregivers should be cognizant of the extensive meaning of spirituality and of the strategies (particularly non-religious measures) to promote it, which will enrich and supplement the process of conservation of dignity.

‘Rootedness’ emphasizes comfort, security, belonging, and emotional connection with families, relatives, or friends [42, 43]. A sense of growth is experienced by patients who alter stressful events into situations with a positive meaning [44]. ‘Rootedness’ and ‘growth’ atmosphere were considered as the environmental factor related to dignity, with ‘rootedness’ comprising the physical atmosphere of ‘homeness’ and the psychological environment of ‘belonging’. Our study revealed that providing the patients with a ‘homeness’ atmosphere, such as a private, aesthetic, comfortable, and particularly ‘homelike’ room, contributed to maintaining their dignity. Studies in Iran and other parts of the world have corroborated these findings and described well-decorated and privacy-assisted living conditions with particularly ‘homelike’ and emotional warmth as prominent factors in preserving patient dignity [61–64]. Our findings showed that patients regarded a ‘homelike’ environment as more important than a basic physical environment (private, aesthetic, and comfortable). The assumption that ‘home’ offers a safe environment is widely reported in the literature, with safety as a recurring and indispensable factor that creates a sense of place-belongingness [65–67]. This is in line with our concept of ‘belonging’ as a part of the psychological environment. Some studies have suggested that the sense of belonging resulted from a ‘homelike’ atmosphere and intimate connections with family, relatives, friends, or others [43, 67]. Moreover, belongingness was shown to provide a feeling of security and trust and a stronger sense of cohesion that could protect against frailty and preserve the sense of dignity [59, 60].

Positive expression is a positive life change and perception of growth, which elevates an individual’s sense of worth and value [44]. Positive behaviors such as expressing thanks and being courteous were cited by caregivers as factors that maintained patient dignity. Furthermore, positive actions may facilitate an intimate and reciprocal relationship that may foster a sense of belonging and elevate patient dignity [68]. Moreover, a positive approach was found to be a product of meaning-making processes and found to gain value from adversity [44]. For example, care needing could be stressful for patients, but it might translate into a positive aspect, such as the appreciation of life, via the meaning-making processes. This development of a positive meaning constitutes the core measurement of dignity therapy [69]. Therefore, caregivers should focus on not only providing patients with a basic physical environment but also on enabling a ‘homelike’ physical environment as well as a ‘belonging’ psychological environment. Additionally, patients should be encouraged to derive meaning from their situation and attempt to obtain value and growth from their stressful events.

Our findings revealed that patients required social support to maintain a balanced state, and excessive care burden may impair their dignity and relationship with their caregivers. In this study, the excessive care burden caused by patients’ health status greatly strained caregivers. This could increase the patients’ feelings of guilt and uselessness, thereby lowering their dignity [36]. However, caregivers reported that they did not routinely discuss their distress or desires with their patients to relieve their patient’s psychological burden [36]. Moreover, a previous study revealed that the enormous care pressure and repressed psychological pressure led to a poor caregiver-care recipient relationship that was detrimental to dignity preservation [70]. Additionally, our research found that inadequate medical information harmed the role adjustment of caregivers and patients and increased the psychological burden among both patients and caregivers. Similarly, another study also indicated that informational support from the medical staff was an effective way to facilitate their adjustment [71]. Furthermore, our findings revealed that inadequate medical information caused illness-related uncertainty, which was linked to patients’ sense of losing control and endangered their dignity [49]. Thus, we can infer that alleviating the care burden, clarifying the relationship or role of the caregivers and patients, and providing adequate medical information are key factors in ameliorating the caregiver-care receiver relationship and patients’ feelings of worthlessness and psychological burden. Finally, adequate social, psychological, and medical information support will help patients adapt to their balanced state and maintain their dignity.

### Implications for theory, research, and practice

Patient dignity is a multidimensional and complex concept. Our study employed Nightingale’s environmental theory, which was conducive to systematically exploring the concept of dignity. Dignity was defined as a multidimensional concept involving individual, physical, psychological, and social environment aspects. First, an individual with dignity should be viewed as an integrated person. Integrity includes a natural life and spiritual life that feature autonomy preservation, role maintenance, and spiritual health. Second, the environment of dignity should incorporate a ‘rootedness’ and ‘growth’ atmosphere comprising a physical environment of ‘homeness’ and a psychological environment of ‘belonging’ and ‘positive meaning’. Third, social support is necessary to maintain one’s balanced state. Thus, dignity can be considered to originate from its inherent dimension, imbuing a longing for being treated as an integrated person to gain self-worth and promote self-respect, as well as instilling a desire for developing belongingness and an atmosphere with a ‘positive meaning’ to achieve self-value and self-growth. Furthermore, external dimensions, such as social support, may help promote one’s dignity. Our study supplements previous studies that mainly defined dignity according to its external dimension [9, 13–15]. Additionally, our findings supported the notion that the caregiver-care recipient relationship was present at the core of the multidimensions of dignity (person, environment, and society) and served as a key factor connecting the dimensions of person, environment, and society. For example, the first category of integrated person indicated that a balance should be maintained between providing care and receiving care. The second category of ‘rootedness’ and ‘growth’ atmosphere suggested a close connection experienced by patients. The third category of balanced state emphasized the role adjustment between patients and caregivers. Further studies should explore the mechanism of relationships in the context of dignity preservation. Moreover, our study results added to the concept of dignity from the perspective of caregivers who provided care for individuals directly, which might be of clinical and research significance when evaluated against previous studies. Compared with prior research conducted from the perspective of patients and medical professionals, caregivers, patients, and medical professionals highlighted that the participation of patients in the caring process might increase their sense of worth and help maintain their dignity [9]. Additionally, all three groups underscored the contradiction between providing care and maintaining patient autonomy [9]. Further research is therefore required to assess the balance of care behavior to support patient autonomy and self-worth, as well as to provide appropriate assistance. Furthermore, divergence in perspectives was evident between caregivers, patients, and medical professionals, with a notable difference being that nurses stressed the need for respect in the work environment while caregivers emphasized an intimate ‘homeness’ environment to maintain dignity. A particular perspective may be suited to a certain situation, suggesting that dignity may be related to the context and should be investigated across different situations. The other difference is that nurses did not mention spiritual concerns, whereas caregivers recognized spiritual needs for promoting dignity but restricted it to the religious domain. This indicated that spirituality was likely to be neglected and should be further investigated. Finally, the common dignity-conserving care model framework and specified frameworks that are appropriate for each group, including caregivers, patients, or medical professionals, can be developed based on those different perspectives.

### Strengths and limitations

This meta-synthesis study has certain strengths that deserve mention. First, considering that caregivers undertake the main care responsibilities, it is important to provide direct evidence relating to preserving patient dignity from the perspective of caregivers. Second, this research has added new findings illustrating the differing views of patients and nurses. In particular, patients might be reluctant to share spiritual concerns with their nurses; however, they were willing to communicate such concerns with their caregivers. Third, the study results are based on the concept of people, environment, and society and are supported by the framework of Nightingale’s environmental theory, which may help systematically elucidate the complex concept of dignity via its multidimensions. Finally, our study produced a valid framework to reflect on the existing care model and indicated the need for in-depth research to construct an effective dignity care model.

Our meta-synthesis analysis has certain limitations that should be considered. The first limitation is that our literature search was confined to articles published in English, and noteworthy research written in other languages might have been ignored. The second limitation relates to using electronic scientific databases to identify eligible studies. Thus, some relevant articles indexed in other databases might have been neglected. The third limitation is that original data was not accessed, and therefore, any original bias in the primary studies could have led to bias in the synthesis. Finally, another limitation is that all the included studies had only conducted research at one time point. Therefore, longitudinal studies are required to investigate the changes in caregivers’ perspectives on dignity preservation over time.

## Conclusion

The model of patient dignity from the caregivers’ perspective is based on a multidimensional structure of human dignity. Our study offers evidence for understanding patient dignity from the caregivers’ perspective and adds supporting information for improving nursing outcomes in patients. Our findings indicate that dignity is rooted in its inherent dimension, emphasizing treating patients as integrated individuals to preserve their self-worth and self-respect and developing belongingness and an atmosphere with a positive meaning to facilitate them to achieve self-value and self-growth. Furthermore, external dimensions, such as social support, may promote one’s dignity.

## Data Availability

All data generated or analyzed during this study are included in this published article.

## References

[1] Galvin K, Todres L (2015). Dignity as honor-wound: an experiential and relational view. J Eval Clin Pract.

[2] United Nations (UN). Universal Declaration of Human Rights 1948. 2019. https://www.un.org/en/universaldeclaration-human-rights/. Accessed 4 Nov 2019.

[3] International Council of Nurses. The ICN Code of Ethics for Nurses 2012. 2019. https://www.aynla.org/2012/12/icn-code-of-ethics-for-nurses-2012/. Accessed 4 Nov 2019. 10.1177/096973300100800409 16004091

[4] Winter SF, Winter SF (2018). Human dignity as leading principle in public health ethics: A multi-case analysis of 21st century German health policy decisions. Int J Health Policy Manag.

[5] Helse-og Omsorgsdepartementet [Norwegian Ministry of Health and Care Services]. Lov om kommunale helse-ogomsorgstjenester m.m. (helse-og omsorgstjenesteloven). 2021. https://lovdata.no/dokument/NL/lov/2011-06-24-30?q¼helse. Accessed 1 July 2021. Accessed 1 Aug 2019.

[6] Finish Ministry of Social Affairs and Health. Act on supporting the functional capacity of the older population and on social and health care services for older persons. 2019. https://www.finlex.fi/fi/laki/kaannokset/.2012/en20120980_20120980.pdf. Accessed 1 Aug 2019.

[7] Sundheds-og ældreministeriet [Danish Ministry of Health and the Aged]. Bekendtgørelse om værdighedspolitikker for ældreplejen [Executive order on dignity policies for the elderly]. 2019. https://www.retsinformation.dk/Forms/R0710.aspx?id¼206695. Accessed 1 Aug 2019.

[8] Socialstyrelsen [Swedish National Board of Health and Welfare]. Socialstyrelsens allma¨nna ra°d om va¨rdegrundeni socialtja¨nstens omsorg om a¨ ldre. Available from: https://www.socialstyrelsen.se/globalassets/sharepoint-okument/artikelkatalog/foreskrifter-och-allmanna-rad/2012-2-Nurs Ethics. Accessed 20 September 2019.

[9] Šaňáková S, Čáp J (2019). Dignity from the nurses' and older patients' perspective: A qualitative literature review. Nurs Ethics.

[10] Chochinov HM (2012). Dignity therapy.

[11] Nordenfelt L (2009). The concept of dignity.

[12] Kadivar M, Mardani-Hamooleh M, Kouhnavard M (2018). Concept analysis of human dignity in patient care: Rodgers' evolutionary approach. J Med Ethics Hist Med.

[13] Pols J, Pasveer B, Willems D (2018). The particularity of dignity: relational engagement in care at the end of life. Med Health Care Philos.

[14] Leget C (2013). Analyzing dignity: a perspective from the ethics of care. Med Health Care Philos.

[15] Tranvag O, Petersen KA, Naden D (2015). Relational interactions preserving dignity experience: perceptions of persons living with dementia. Nurs Ethics.

[16] Li YC, Feng YH, Ma SC, Wang HH (2023). Dignity and Related Factors in Patients with Cancer: A Cross-Sectional Study. Asian Nurs Res (Korean Soc Nurs Sci).

[17] Bidabadi FS, Yazdannik A, Zargham-Boroujeni A (2019). Patient's dignity in intensive care unit: A critical ethnography. Nurs Ethics.

[18] Torabizadeh C, Jafari S, Momennasab M (2021). Patient's Dignity: Viewpoints of Patients and Nurses in Hospitals. Hosp Top.

[19] Butkevičienė R, Kuznecovienė J, Harrison D, Peičius E, Urbonas G, Astromskė K, Kalėdienė R (2021). Being Heard: A Qualitative Study of Lithuanian Health Care Professionals' Perceptions of Dignity at the End-of-Life. Medicina (Kaunas).

[20] Nåden D, Rehnsfeldt A, Råholm MB, Lindwall L, Caspari S, Aasgaard T, Slettebø Å, Sæteren B, Høy B, Lillestø B, Heggestad AK, Lohne V (2013). Aspects of indignity in nursing home residences as experienced by family caregivers. Nurs Ethics.

[21] Devik SA, Enmarker I, Wiik GB, Hellzèn O (2013). Meanings of being old, living on one's own and suffering from incurable cancer in rural Norway. Eur J Oncol Nurs.

[22] van der Geugten W, Goossensen A (2020). Dignifying and undignifying aspects of care for people with dementia: a narrative review. Scand J Caring Sci.

[23] Hegge M (2013). Nightingale's environmental theory. Nurs Sci Q.

[24] Nightingale F (1980). Notes on Nursing, what it is and what it is not.

[25] Tong A, Flemming K, McInnes E (2012). Enhancing transparency in reporting the synthesis of qualitative research: ENTREQ. BMC Med Res Methodol.

[26] Critical Appraisal Skills Programme. CASP Qualitative checklist. 2018. https://casp-uk.net/casp-tools-checklists/. Accessed March 2022.

[27] Thomas J, Harden A (2008). Methods for the thematic synthesis of qualitative research in systematic reviews. BMC Med Res Methodol.

[28] Butler A, Hall H, Copnell B (2016). A Guide to Writing a Qualitative Systematic Review Protocol to Enhance Evidence-Based Practice in Nursing and Health Care. Worldviews Evid Based Nurs.

[29] Dixon-Woods M, Agarwal S, Jones D, Young B, Sutton A (2005). Synthesising qualitative and quantitative evidence: a review of possible methods. J Health Serv Res Policy.

[30] Eilertsen G, Ormstad H, Kirkevold M (2013). Experiences of poststroke fatigue: a qualitative meta-synthesis. J Adv Nurs.

[31] Graneheim UH, Lundman B (2004). Qualitative content analysis in nursing research: concepts, procedures and measures to achieve trustworthiness. Nurse Educ Today.

[32] Kalis A, Schermer MHN, van Delden JMJ (2005). Ideals regarding a good life for nursing home residents with dementia: views of professional caregivers. Nurs Ethics.

[33] Anderson JG, Bartmess M, Hundt E, Jacelon C (2021). "A little bit of their souls": Investigating the concept of dignity for people living with dementia using caregivers' blogs. J Fam Nurs.

[34] Tehranineshat B, Rakhshan M, Torabizadeh C, Fararouei M (2020). Patient dignity in Iranian clinical care settings as perceived by physicians, caregivers, and patients. J Multidiscip Healthc.

[35] Gharibian Adra M, Hopton J, Keady J (2015). Constructing the meaning of quality of life for residents in care homes in the Lebanon: perspectives of residents, staff and family. Int J Older People Nurs.

[36] Melin-Johansson C, Axelsson B, Danielson E (2007). Caregivers' perceptions about terminally ill family members' quality of life. Eur J Cancer Care (Engl).

[37] Caspari S, Lohne V, Rehnsfeldt AW, Sæteren B, Slettebø A, Tolo Heggestad AK, Lillestø B, Høy B, Råholm MB, Lindwall L, Aasgaard T, Nåden D (2014). Dignity and existential concerns among nursing homes residents from the perspective of their relatives. Clin Nurs Stud.

[38] Rehnsfeldt A, Lindwall L, Lohne V, Lillestø B, Slettebø A, Tolo Heggestad AK (2014). The meaning of dignity in nursing home care as seen by relatives. Nurs Ethics.

[39] Lou C, Lou K, Ridley J (2021). Exploring the meaning of dignity at end of life for Chinese Canadians caregivers: A qualitative cross-cultural study. Palliat Med.

[40] Sastrawan S, Newton JM, Malik G (2019). Nurses' integrity and coping strategies: An integrative review. J Clin Nurs.

[41] Widäng I, Fridlund B, Mårtensson J (2008). Women patients’ conceptions of integrity within health care: a phenomenographic study. J Adv Nurs.

[42] Andersson M, Hallberg IR, Edberg AK (2008). Old people receiving municipal care, their experiences of what constitutes a good life in the last phase of life: a qualitative study. Int J Nurs Stud.

[43] Söderman T, Östlund U, Werkander Harstäde C, Blomberg K (2020). Dignity-conserving care for persons with palliative care needs - identifying outcomes studied in research: An integrative review. Palliat Support Care.

[44] Park CL, Helgeson VS (2006). Introduction to the special section: growth following highly stressful life events–current status and future directions. J Consult Clin Psychol.

[45] Sanz-Osorio MT, Sastre-Rus M, Monistrol O, Pérez Criado M, Vallès V, Escobar-Bravo MA (2023). Humanization of care in acute psychiatric hospitalization units: A scoping review. J Psychiatr Ment Health Nurs.

[46] Callan VJ, Terry DJ, Schweitzer R (1994). Coping resources, coping strategies and adjustment to organizational change: direct or buffering effects?. Empl Adjust to Organ Chang.

[47] Deci EL, Ryan RM (2012). Self-determination theory in health care and its relations to motivational interviewing: a few comments. Int J Behav Nutr Phys Act.

[48] Nathanson E (2017). Native voice, self-concept, and the moral case for personalized voice technology. Disabil Rehabil.

[49] Ryan RM, Deci EL (2017). Self-Determination Theory: Basic Psychological Needs in Motivation, Development, and Wellness.

[50] Vincensi BB (2019). Interconnections: Spirituality, spiritual care, and patient-centered care. Asia Pac J Oncol Nurs.

[51] Bożek A, Nowak PF, Blukacz M (2020). The relationship between spirituality, health-related behavior, and psychological well-being. Front Psychol.

[52] Cleland J, Hutchinson C, Khadka J, Milte R, Ratcliffe J (2021). What defines quality of care for older people in aged care? A comprehensive literature review. Geriatr Gerontol Int.

[53] Harstäde CW, Blomberg K, Benzein E, Östlund U (2018). Dignity-conserving care actions in palliative care: an integrative review of Swedish research. Scand J Caring Sci.

[54] Iranmanesh S, Tirgari B, Cheraghi MA (2012). Developing and testing a spiritual care questionnaire in the Iranian context. J Relig Health.

[55] Kaddourah B, Abu-Shaheen A, Al-Tannir M (2018). Nurses’ perceptions of spirituality and spiritual care at five tertiary care hospitals in Riyadh, Saudi Arabia: A cross-sectional study. Oman Med J.

[56] Rego F, Nunes R (2019). The interface between psychology and spirituality in palliative care. J Health Psychol.

[57] Evangelista CB, Lopes ME, Costa SF, Batista PS, Batista JB, Oliveira AM (2016). Palliative care and spirituality: an integrative literature review. Rev Bras Enferm.

[58] Holmes C, Kim-Spoon J (2016). Why are religiousness and spirituality associated with externalizing psychopathology? A literature reviews. Clin Child Fam Psychol Rev.

[59] Carey LB, Willis MA, Krikheli L, O'Brien A (2015). Religion, health, and confidentiality: an exploratory review of the role of chaplains. J Relig Health.

[60] Steinhorn DM, Din J, Johnson A (2017). Healing, spirituality, and integrative medicine. Ann Palliat Med.

[61] Naderi Z, Gholamzadeh S, Zarshenas L, Ebadi A (2019). Hospitalized elder abuse in Iran: a qualitative study. BMC Geriatr.

[62] Ferri P, Muzzalupo J, Di Lorenzo R (2015). Patients’ perception of dignity in an Italian general hospital: a cross-sectional analysis. BMC Health Serv Res..

[63] Baillie L, Ford P, Gallagher A, Wainwright P (2009). Nurses' views on dignity in care. Nurs Older People.

[64] Fleming A, Kydd A, Stewart S (2017). Care homes: The developing ideology of a homelike place to live. Maturitas.

[65] Ling J, Payne S, Connaire K, McCarron M (2016). Parental decision-making on utilisation of out-of-home respite in children's palliative care: findings of qualitative case study research - a proposed new model. Child Care Health Dev.

[66] Price J, McCloskey S, Brazil K (2018). The role of hospice in the transition from hospital to home for technology-dependent children-A qualitative study. J Clin Nurs.

[67] Dunbar H, Carter B, Brown J (2019). Coming 'Home': Place bonding for parents accessing or considering hospice based respite. Health Place.

[68] Oser TK, Oser SM, Parascando JA, Hessler-Jones D, Sciamanna CN, Sparling K, Nease D, Litchman ML (2020). Social Media in the Diabetes Community: a Novel Way to Assess Psychosocial Needs in People with Diabetes and Their Caregivers. Curr Diab Rep.

[69] Bluck S, Mroz EL, Wilkie DJ, Emanuel L, Handzo G, Fitchett G, Chochinov HM, Bylund CL (2022). Quality of Life for Older Cancer Patients: Relation of Psychospiritual Distress to Meaning-Making During Dignity Therapy. Am J Hosp Palliat Care.

[70] Bidwell JT, Lyons KS, Lee CS (2017). Caregiver well-being and patient outcomes in heart failure: A meta-analysis. J Cardiovasc Nurs.

[71] Whitlatch CJ, Orsulic-Jeras S (2018). Meeting the informational, educational, and psychosocial support needs of persons living with dementia and their family caregivers. Gerontologist.

